# Supplementary material for: A Likelihood Approach for Real-Time Calibration of Stochastic Compartmental Epidemic Models
Source: PLoS Comput Biol. 2017 Jan 17;13(1):e1005257. doi: 10.1371/journal.pcbi.1005257 (PMC5240920; doi:10.1371/journal.pcbi.1005257)
Supplement: S1 File — (TAR.GZ) [file pcbi.1005257.s014.tar.gz › HSPH_Online-SI-Revision/Additions/EnKF08/n100-extreme-SEITR/n100-extreme-SEITR_EnKF2_table8.pdf]

1	newly 3 weeks spec	108.	3634.62	{426., 14114.}	3273.19	1.09591	newly 3 weeks cumul	716.	13893.4	{2638., 43587.}	1843.74	0.970627	newly 5 weeks spec	52.	2031.69	{78., 9568.}	3818.67	1.04319	newly 5 weeks cumul	854.	18721.1	{3416., 74788.}	2095.55	0.99871	newly 43 weeks cumul	939.	22884.4	{3497., 90004.}	2340.51	1.0288
2	newly 3 weeks spec	102.	1432.18	{81., 7050.}	1315.43	0.788271	newly 3 weeks cumul	593.	12378.5	{1719., 49183.}	1989.38	0.900703	newly 5 weeks spec	36.	273.53	{6., 507.}	682.028	0.738715	newly 5 weeks cumul	673.	13234.3	{1937., 62625.}	1868.39	0.893724	newly 50 weeks cumul	712.	13952.1	{2042., 67461.}	1861.47	0.902778
3	newly 3 weeks spec	169.	1215.93	{17., 401.}	694.337	0.580649	newly 3 weeks cumul	920.	4542.53	{217., 2515.}	418.084	0.409287	newly 5 weeks spec	52.	528.51	{0., 159.}	1025.02	1.09784	newly 5 weeks cumul	1072.	5934.84	{217., 3250.}	478.196	0.399488	newly 52 weeks cumul	1137.	7252.66	{219., 3362.}	562.906	0.409536
4	newly 3 weeks spec	137.	2615.79	{253., 7226.}	1820.69	1.09089	newly 3 weeks cumul	790.	15957.9	{5731., 27241.}	1920.47	1.11719	newly 5 weeks spec	41.	1751.74	{13., 8940.}	4203.56	1.28655	newly 5 weeks cumul	909.	19801.1	{6789., 43268.}	2078.34	1.1539	newly 50 weeks cumul	985.	26928.	{7412., 86205.}	2633.81	1.24454
5	newly 3 weeks spec	34.	762.1	{0., 252.}	2272.59	1.90335	newly 3 weeks cumul	171.	8103.61	{313., 32728.}	4639.95	0.92576	newly 5 weeks spec	11.	392.95	{0., 42.}	3619.91	1.70853	newly 5 weeks cumul	195.	9088.84	{313., 36942.}	4561.95	0.885178	newly 52 weeks cumul	201.	9615.69	{313., 37171.}	4684.96	0.877221
6	newly 3 weeks spec	5.	1437.17	{199., 2353.}	28645.	2.14395	newly 3 weeks cumul	60.	8258.92	{2513., 24862.}	13664.9	1.86072	newly 5 weeks spec	2.	548.71	{22., 1317.}	27337.5	2.03356	newly 5 weeks cumul	63.	9680.69	{2941., 24862.}	15266.2	1.915	newly 51 weeks cumul	64.	11117.2	{3036., 27582.}	17270.7	1.96845
7	newly 3 weeks spec	469.	894.49	{192., 2543.}	142.258	0.34876	newly 3 weeks cumul	2142.	7004.7	{1197., 17629.}	270.317	0.372054	newly 5 weeks spec	221.	357.23	{57., 628.}	127.986	0.375757	newly 5 weeks cumul	2717.	7899.05	{1553., 22593.}	233.867	0.352992	newly 43 weeks cumul	3226.	8695.77	{1921., 24140.}	212.627	0.338289
8	newly 3 weeks spec	225.	1266.45	{69., 1768.}	491.729	0.657313	newly 3 weeks cumul	1088.	7959.92	{346., 10983.}	651.04	0.779772	newly 5 weeks spec	80.	532.7	{6., 1058.}	616.2	0.866185	newly 5 weeks cumul	1321.	9291.65	{441., 11314.}	622.503	0.768021	newly 50 weeks cumul	1472.	10577.	{532., 13674.}	637.182	0.776541
9	newly 3 weeks spec	19.	1944.96	{67., 7485.}	10139.7	1.59061	newly 3 weeks cumul	167.	13691.3	{1834., 57616.}	8100.53	1.56178	newly 5 weeks spec	5.	481.75	{5., 1481.}	9546.2	1.53018	newly 5 weeks cumul	179.	15104.8	{1858., 62534.}	8340.61	1.57933	newly 52 weeks cumul	188.	15869.1	{1858., 63713.}	8343.24	1.58305
10	newly 3 weeks spec	613.	723.4	{0., 1683.}	136.529	2.19456	newly 3 weeks cumul	2661.	23036.2	{1841., 80984.}	776.017	0.650803	newly 5 weeks spec	260.	786.42	{0., 983.}	318.515	2.16889	newly 5 weeks cumul	3324.	24573.3	{2439., 80984.}	647.003	0.622788	newly 48 weeks cumul	3949.	30141.3	{3634., 81371.}	669.9	0.655016
11	newly 3 weeks spec	448.	4441.27	{167., 17903.}	924.614	0.779452	newly 3 weeks cumul	2137.	15678.2	{687., 49024.}	670.895	0.758184	newly 5 weeks spec	237.	2627.53	{48., 11485.}	1039.16	0.750784	newly 5 weeks cumul	2689.	21776.9	{868., 78506.}	745.427	0.761502	newly 47 weeks cumul	3221.	29290.8	{1419., 111443.}	837.618	0.758754
12	newly 3 weeks spec	209.	1796.17	{55., 5446.}	782.1	0.711477	newly 3 weeks cumul	1086.	7205.01	{405., 13975.}	580.959	0.666023	newly 5 weeks spec	68.	1331.71	{6., 5534.}	1903.54	1.02463	newly 5 weeks cumul	1294.	10051.2	{410., 25169.}	694.563	0.703043	newly 48 weeks cumul	1442.	16002.8	{540., 55329.}	1026.79	0.760637
13	newly 3 weeks spec	300.	2828.54	{0., 9860.}	919.62	1.35257	newly 3 weeks cumul	1508.	20429.9	{4170., 53364.}	1263.53	0.947106	newly 5 weeks spec	131.	1394.97	{0., 4602.}	1077.09	1.80907	newly 5 weeks cumul	1818.	23788.3	{4867., 65043.}	1217.97	0.925127	newly 49 weeks cumul	2109.	27239.1	{5211., 86057.}	1201.6	0.913901
14	newly 3 weeks spec	681.	2976.62	{0., 16329.}	473.119	1.43602	newly 3 weeks cumul	2981.	9515.33	{151., 43450.}	302.1	0.638227	newly 5 weeks spec	331.	1020.1	{0., 3782.}	356.332	1.90913	newly 5 weeks cumul	3718.	12418.3	{153., 59993.}	326.623	0.695226	newly 42 weeks cumul	4474.	14021.1	{153., 79347.}	314.15	0.742205
15	newly 3 weeks spec	238.	3892.5	{24., 19670.}	1568.12	0.93265	newly 3 weeks cumul	1233.	17123.6	{269., 79110.}	1318.98	0.857645	newly 5 weeks spec	100.	1522.3	{12., 6974.}	1457.86	0.942971	newly 5 weeks cumul	1479.	21062.1	{294., 92912.}	1353.56	0.871595	newly 43 weeks cumul	1667.	24515.3	{303., 94246.}	1399.11	0.889227
16	newly 3 weeks spec	233.	1000.91	{120., 1307.}	346.76	0.52401	newly 3 weeks cumul	1269.	6910.13	{599., 7380.}	464.827	0.612842	newly 5 weeks spec	91.	424.23	{32., 623.}	399.484	0.545723	newly 5 weeks cumul	1497.	7962.05	{760., 7897.}	450.325	0.606311	newly 46 weeks cumul	1647.	9375.57	{920., 9372.}	485.026	0.618535
17	newly 3 weeks spec	1.	2190.38	{7., 7896.}	218938.	2.12789	newly 3 weeks cumul	62.	11320.	{1846., 36795.}	18158.	1.77963	newly 5 weeks spec	0.1	555.46	{0., 1006.}	555438.	1.86984	newly 5 weeks cumul	64.	12928.5	{1851., 39748.}	20100.7	1.80132	newly 52 weeks cumul	64.	14020.5	{1853., 40428.}	21807.	1.82503
18	newly 3 weeks spec	93.	1482.83	{42., 3125.}	1511.52	0.80989	newly 3 weeks cumul	605.	8654.03	{606., 29682.}	1339.95	0.744867	newly 5 weeks spec	53.	560.67	{9., 2164.}	994.321	0.852807	newly 5 weeks cumul	722.	10105.2	{677., 39035.}	1309.08	0.747282	newly 51 weeks cumul	795.	11510.	{789., 43645.}	1356.73	0.776704
19	newly 3 weeks spec	465.	648.05	{0., 1689.}	123.822	1.25394	newly 3 weeks cumul	2284.	14513.5	{209., 50023.}	564.315	0.739579	newly 5 weeks spec	217.	207.52	{0., 622.}	94.5899	1.28119	newly 5 weeks cumul	2812.	15085.9	{257., 50023.}	465.204	0.693085	newly 46 weeks cumul	3336.	15383.7	{332., 50023.}	389.605	0.640678
20	newly 3 weeks spec	75.	2144.4	{202., 5669.}	2759.2	1.06989	newly 3 weeks cumul	401.	7818.34	{611., 18572.}	1850.73	0.927791	newly 5 weeks spec	14.	1390.51	{176., 4529.}	9832.21	1.59884	newly 5 weeks cumul	459.	10945.8	{1043., 28306.}	2284.84	1.01217	newly 51 weeks cumul	486.	17459.2	{3816., 57547.}	3492.42	1.24763
21	newly 3 weeks spec	38.	130.16	{0., 437.}	380.947	1.92177	newly 3 weeks cumul	270.	2087.87	{770., 2499.}	674.53	0.675611	newly 5 weeks spec	12.	46.97	{0., 147.}	440.083	1.77752	newly 5 weeks cumul	303.	2211.99	{770., 2945.}	631.297	0.642711	newly 52 weeks cumul	316.	2313.53	{770., 3229.}	633.402	0.633215
22	newly 3 weeks spec	162.	3366.85	{44., 17345.}	2031.06	0.644039	newly 3 weeks cumul	806.	13351.5	{954., 54827.}	1560.91	0.641849	newly 5 weeks spec	58.	592.15	{1., 1099.}	1029.36	0.99573	newly 5 weeks cumul	951.	15507.9	{997., 74441.}	1536.36	0.608077	newly 48 weeks cumul	1042.	15825.3	{1014., 77779.}	1425.47	0.580248
23	newly 3 weeks spec	22.	941.22	{21., 2364.}	4190.36	1.22916	newly 3 weeks cumul	142.	13372.9	{1040., 30483.}	9317.56	1.73249	newly 5 weeks spec	3.	157.38	{0., 549.}	5182.	1.31372	newly 5 weeks cumul	156.	13908.1	{1044., 33683.}	8815.46	1.70919	newly 49 weeks cumul	161.	14020.9	{1044., 34059.}	8608.6	1.69889
24	newly 3 weeks spec	51.	2095.	{144., 1384.}	4014.24	1.21909	newly 3 weeks cumul	312.	10395.7	{1796., 16825.}	3231.96	1.21947	newly 5 weeks spec	15.	989.99	{17., 740.}	6509.53	1.24461	newly 5 weeks cumul	360.	12825.4	{2476., 23921.}	3462.62	1.23045	newly 51 weeks cumul	369.	15122.8	{2552., 24111.}	3998.33	1.27784
25	newly 3 weeks spec	160.	2086.36	{138., 3487.}	1214.25	0.723466	newly 3 weeks cumul	871.	7847.4	{591., 14295.}	814.512	0.657296	newly 5 weeks spec	60.	1154.59	{60., 1826.}	1832.58	0.822529	newly 5 weeks cumul	1024.	10561.1	{719., 18599.}	943.824	0.686955	newly 47 weeks cumul	1086.	13591.4	{845., 23281.}	1161.99	0.74094
26	newly 3 weeks spec	96.	1070.75	{15., 2773.}	1055.99	0.686014	newly 3 weeks cumul	682.	7245.71	{1709., 22455.}	967.696	0.794498	newly 5 weeks spec	33.	231.36	{0., 556.}	705.333	1.15892	newly 5 weeks cumul	784.	7989.56	{1720., 27778.}	924.536	0.759963	newly 45 weeks cumul	878.	8224.	{1720., 30640.}	842.394	0.721707
27	newly 3 weeks spec	572.	2290.75	{475., 5504.}	311.533	0.335186	newly 3 weeks cumul	2438.	10145.9	{3163., 38459.}	321.628	0.408169	newly 5 weeks spec	284.	994.35	{69., 910.}	287.75	0.38037	newly 5 weeks cumul	3101.	12697.2	{3921., 45495.}	315.694	0.392955	newly 45 weeks cumul	3756.	14483.2	{4492., 49474.}	292.614	0.378777
28	newly 3 weeks spec	8.	996.91	{17., 1399.}	12363.4	1.07469	newly 3 weeks cumul	54.	3753.68	{360., 4220.}	6853.	1.27931	newly 5 weeks spec	1.	510.85	{1., 541.}	50999.	1.18192	newly 5 weeks cumul	61.	5000.6	{367., 6810.}	8099.48	1.27084	newly 52 weeks cumul	63.	5831.86	{367., 10703.}	9158.7	1.27807
29	newly 3 weeks spec	378.	1346.98	{43., 2171.}	325.233	0.542582	newly 3 weeks cumul	1806.	12069.6	{818., 26532.}	602.126	0.498555	newly 5 weeks spec	145.	405.07	{0., 390.}	282.462	0.99446	newly 5 weeks cumul	2196.	13171.6	{863., 26534.}	535.043	0.485463	newly 49 weeks cumul	2473.	14327.9	{869., 60305.}	514.928	0.486418
30	newly 3 weeks spec	146.	1561.5	{70., 899.}	993.151	0.477385	newly 3 weeks cumul	831.	10398.	{546., 6761.}	1163.71	0.615665	newly 5 weeks spec	47.	503.95	{9., 315.}	1036.91	0.607847	newly 5 weeks cumul	975.	11740.8	{579., 7293.}	1118.48	0.601453	newly 52 weeks cumul	1051.	12607.	{601., 7405.}	1115.05	0.595528
31	newly 3 weeks spec	292.	1230.76	{50., 2059.}	350.527	0.540258	newly 3 weeks cumul	1423.	6540.02	{677., 10310.}	374.533	0.596036	newly 5 weeks spec	119.	677.67	{3., 885.}	539.303	0.785341	newly 5 weeks cumul	1719.	8117.02	{690., 16892.}	387.866	0.58693	newly 42 weeks cumul	1955.	10045.	{723., 18548.}	429.81	0.586263
32	newly 3 weeks spec	532.	3227.41	{28., 12042.}	566.6	0.696994	newly 3 weeks cumul	2380.	9959.27	{474., 28514.}	367.291	0.584212	newly 5 weeks spec	254.	2491.98	{2., 11890.}	944.37	1.0118	newly 5 weeks cumul	3005.	15389.1	{550., 53559.}	459.762	0.608012	newly 48 weeks cumul	3596.	23310.9	{566., 93028.}	590.184	0.632536
33	newly 3 weeks spec	65.	571.27	{0., 1055.}	857.954	1.05178	newly 3 weeks cumul	390.	4066.26	{542., 8994.}	945.949	0.578243	newly 5 weeks spec	28.	293.88	{0., 290.}	1067.57	1.30869	newly 5 weeks cumul	468.	4741.8	{565., 14706.}	917.671	0.543592	newly 52 weeks cumul	508.	5670.67	{565., 16722.}	1021.31	0.530618
34	newly 3 weeks spec	107.	1297.08	{15., 2679.}	1180.43	0.664865	newly 3 weeks cumul	548.	12536.9	{2050., 34213.}	2188.49	0.943419	newly 5 weeks spec	35.	148.58	{0., 262.}	466.743	1.57413	newly 5 weeks cumul	648.	13109.9	{2051., 41566.}	1924.05	0.884331	newly 48 weeks cumul	692.	13197.6	{2051., 42569.}	1808.16	0.858366
35	newly 3 weeks spec	10.	1717.92	{2., 4335.}	17109.6	1.23309	newly 3 weeks cumul	81.	6229.8	{483., 13287.}	7599.36	1.32684	newly 5 weeks spec	0.1	702.55	{0., 1377.}	702522.	1.89439	newly 5 weeks cumul	82.	8075.72	{561., 18897.}	9756.2	1.37441	newly 52 weeks cumul	82.	9067.02	{597., 22253.}	10965.	1.41321
36	newly 3 weeks spec	587.	3111.77	{2., 10740.}	472.083	0.876114	newly 3 weeks cumul	2638.	18416.1	{3235., 48652.}	607.881	0.657261	newly 5 weeks spec	275.	1353.15	{0., 2178.}	475.502	1.28514	newly 5 weeks cumul	3306.	21737.9	{3235., 64081.}	567.597	0.637195	newly 48 weeks cumul	3908.	25944.5	{7888., 87999.}	573.426	0.646202
37	newly 3 weeks spec	138.	423.76	{68., 721.}	235.696	0.556197	newly 3 weeks cumul	721.	6979.91	{2832., 9347.}	868.087	0.809671	newly 5 weeks spec	53.	105.35	{2., 199.}	215.491	0.791445	newly 5 weeks cumul	854.	7268.7	{2969., 10135.}	751.136	0.757811	newly 49 weeks cumul	922.	7533.54	{2984., 12854.}	717.087	0.739634
38	newly 3 weeks spec	103.	2544.34	{364., 5999.}	2371.18	1.2027	newly 3 weeks cumul	598.	14613.5	{3266., 28234.}	2344.37	1.20628	newly 5 weeks spec	29.	1162.04	{78., 3738.}	3912.07	1.2678	newly 5 weeks cumul	693.	17428.9	{3715., 33270.}	2415.42	1.22579	newly 46 weeks cumul	752.	21065.8	{3715., 45649.}	2701.64	1.27239
39	newly 3 weeks spec	188.	4129.92	{377., 10998.}	2099.7	0.953748	newly 3 weeks cumul	946.	14905.8	{2615., 44098.}	1477.76	0.938728	newly 5 weeks spec	61.	2448.87	{28., 10572.}	3932.21	1.14991	newly 5 weeks cumul	1112.	20560.6	{3797., 53313.}	1750.91	0.993449	newly 48 weeks cumul	1224.	28112.7	{6180., 87418.}	2198.64	1.07795
40	newly 3 weeks spec	395.	1892.73	{257., 4288.}	396.104	0.530544	newly 3 weeks cumul	1868.	9492.07	{1276., 27431.}	422.947	0.499357	newly 5 weeks spec	218.	861.05	{79., 2006.}	323.353	0.510496	newly 5 weeks cumul	2391.	11612.2	{1534., 29467.}	401.365	0.500612	newly 49 weeks cumul	2892.	13989.4	{1719., 33336.}	400.383	0.519155
41	newly 3 weeks spec	12.	1344.72	{8., 676.}	11118.3	0.858106	newly 3 weeks cumul	121.	4256.65	{281., 4072.}	3417.89	0.978304	newly 5 weeks spec	2.	496.03	{0., 335.}	24765.5	1.21286	newly 5 weeks cumul	135.	5636.97	{307., 4196.}	4075.53	0.964596	newly 52 weeks cumul	137.	6230.49	{315., 4503.}	4447.8	0.972497
42	newly 3 weeks spec	675.	2401.76	{62., 11468.}	321.887	0.601783	newly 3 weeks cumul	2966.	16667.9	{802., 54753.}	481.964	0.490363	newly 5 weeks spec	340.	572.01	{1., 1849.}	177.826	0.938629	newly 5 weeks cumul	3794.	18360.5	{1142., 63064.}	407.901	0.455978	newly 43 weeks cumul	4690.	19471.1	{2059., 64544.}	350.511	0.453019
43	newly 3 weeks spec	545.	723.78	{0., 1455.}	137.64	1.86052	newly 3 weeks cumul	2315.	13741.7	{3659., 36054.}	499.009	0.531285	newly 5 weeks spec	245.	381.31	{0., 695.}	164.38	1.97197	newly 5 weeks cumul	2918.	14659.	{3659., 43648.}	409.899	0.491607	newly 48 weeks cumul	3370.	15453.9	{3659., 43648.}	367.35	0.469324
44	newly 3 weeks spec	215.	1764.68	{32., 1123.}	804.605	0.593138	newly 3 weeks cumul	1211.	6668.75	{1342., 8239.}	458.095	0.399581	newly 5 weeks spec	93.	351.19	{0., 263.}	421.968	1.37499	newly 5 weeks cumul	1475.	7853.91	{1351., 9841.}	441.746	0.352777	newly 48 weeks cumul	1690.	8216.24	{1351., 13305.}	397.993	0.322366
45	newly 3 weeks spec	201.	2480.02	{72., 6963.}	1156.89	0.852653	newly 3 weeks cumul	1070.	18003.9	{3719., 72879.}	1582.61	0.874023	newly 5 weeks spec	90.	633.42	{1., 978.}	656.622	1.06759	newly 5 weeks cumul	1275.	19745.4	{4509., 80262.}	1448.66	0.867697	newly 50 weeks cumul	1419.	22162.3	{5076., 80905.}	1461.82	0.885851
46	newly 3 weeks spec	138.	2430.16	{24., 8879.}	1718.12	0.773885	newly 3 weeks cumul	722.	11863.2	{826., 31358.}	1552.45	0.705641	newly 5 weeks spec	50.	756.5	{0., 2612.}	1502.72	1.27226	newly 5 weeks cumul	861.	13919.4	{1049., 38239.}	1526.04	0.693352	newly 52 weeks cumul	942.	15983.9	{1074., 51602.}	1606.25	0.713963
47	newly 3 weeks spec	224.	3949.81	{694., 12511.}	1663.31	0.965513	newly 3 weeks cumul	1089.	14753.9	{3171., 32005.}	1255.11	0.894545	newly 5 weeks spec	77.	2401.96	{427., 11465.}	3019.43	1.15083	newly 5 weeks cumul	1298.	20262.5	{3870., 56121.}	1461.05	0.944393	newly 47 weeks cumul	1446.	28087.1	{5361., 95714.}	1842.4	1.03144
48	newly 3 weeks spec	86.	2169.22	{217., 7127.}	2429.77	1.13404	newly 3 weeks cumul	493.	12660.8	{1065., 23649.}	2476.84	1.17529	newly 5 weeks spec	18.	841.59	{22., 1766.}	4580.06	1.24702	newly 5 weeks cumul	561.	14779.5	{1575., 39638.}	2542.56	1.18831	newly 50 weeks cumul	581.	16980.1	{2748., 64504.}	2829.65	1.22048
49	newly 3 weeks spec	117.	896.88	{9., 2584.}	706.513	0.884935	newly 3 weeks cumul	638.	17459.8	{3627., 41879.}	2636.76	1.14072	newly 5 weeks spec	40.	404.14	{0., 1695.}	998.8	1.54361	newly 5 weeks cumul	753.	18423.	{3630., 42190.}	2347.01	1.12025	newly 48 weeks cumul	828.	19800.4	{3630., 42192.}	2291.89	1.132
50	newly 3 weeks spec	261.	1305.94	{159., 2151.}	419.257	0.549765	newly 3 weeks cumul	1311.	7506.14	{907., 10261.}	484.818	0.545668	newly 5 weeks spec	104.	552.29	{49., 1001.}	457.779	0.603648	newly 5 weeks cumul	1579.	8895.94	{1063., 11577.}	475.562	0.542526	newly 49 weeks cumul	1754.	10099.6	{1159., 14296.}	487.928	0.549038
